# Fulminant endogenous panophthalmitis caused by *Clostridium septicum* infection

**DOI:** 10.5935/0004-2749.20220076

**Published:** 2023

**Authors:** Alicia Berlanga Díaz, Martim Azevedo González-Oliva, Rafael Hervás, Pablo Gili

**Affiliations:** 1 Department of Ophthalmology, Hospital Universitario Fundación Alcorcón, Madrid, Spain; 2 Department of Internal Medicine, Hospital Universitario Fundación Alcorcón, Madrid, Spain

**Keywords:** Panophthalmitis, Adenocarcinoma, Colonic neoplasms, *Clostridium septicum*, Panoftalmite, Adenocarcinoma, Neoplasias do colo, *Clostridium septicum*

## Abstract

We report an unusual case of fulminant endogenous *Clostridium septicum* panophthalmitis. A 74-year-old male patient presented with sudden amaurosis in the right eye, which in a few hours, evolved into an orbital cellulitis, endophthalmitis, anterior segment ischemia, and secondary perforation of the eye. A complete diagnostic study, which included cranial and orbital contrast-enhanced computed tomography scan, contrast-enhanced magnetic resonance imaging, blood cultures, and complete blood work, were performed. No causal agent was identified. *Clostridium septicum* infection caused fulminant gaseous panophthalmitis. Despite broad-spectrum antibiotic treatment, evisceration of the eyeball was necessary. The extension study showed a colon adenocarcinoma as the origin of the infection. *Clostridium septicum* panophthalmitis is a rare but aggressive orbital infection. This infection warrants the identification of a neoplastic process in the gastrointestinal tract in many cases not previously described.

## Abstract (pt)

Este é o relato de um caso incomum de panoftalmite endógena fulminante por *Clostridium septicum*. Um paciente do sexo masculino, 74 anos, apresentou amaurose súbita no olho direito, que em poucas horas evoluiu para celulite orbitária, endoftalmite, isquemia do segmento anterior e perfuração secundária do olho. Foi realizado um estudo diagnóstico completo, que incluiu uma tomografia computadorizada com contraste cranial e orbital, um exame de ressonância magnética, hemocultura e hemograma completo. Nenhum agente causal foi identificado. A infecção por *Clostridium septicum* causou uma panoftalmite gasosa fulminante. Apesar do tratamento com antibióticos de amplo espectro, foi necessário eviscerar o globo ocular. O estudo de seguimento mostrou um adenocarcinoma de cólon como a origem da infecção. A panoftalmite por *Clostridium septicum* é um tipo raro, mas muito agressivo de infecção orbitária. Essa infecção deve sugerir a busca por um processo neoplásico no trato gastrointestinal, em muitos casos não diagnosticado anteriormente.

## INTRODUCTION

Endophthalmitis is most frequently caused by exogenous factors and occurs after a penetrating wound, corneal ulcer, or periocular infection. On the other hand, endogenous endophthalmitis is relatively uncommon, representing only 2-8% of all endophthalmitis cases. It is caused by microorganisms that reach the eye after a bacteremia secondary to an extraocular focus of infection^(1)^. *Clostridium septicum* is rarely the culprit of such cases, and if so, an extensive search for risk factors must be performed.

## CASE REPORT

A 74-year-old male patient with insulin-dependent type 2 diabetes, high blood pressure, dyslipidemia, ischemic cardiomyopathy, carotid atherosclerosis, and cognitive impairment visited our hospital with a painful eye and sudden confusion. He had a prostatic adenocarcinoma removed a year before and was an implantable cardioverter-defibrillator carrier because of ventricular fibrillation episodes. Cranial computed tomography (CT) and blood tests were performed, and the patient was hospitalized in our stroke unit. After a few hours, he developed amaurosis, a reactive myosis, acute pain, hyperemia, and proptosis in his right eye (RE).

**Figure 1:**
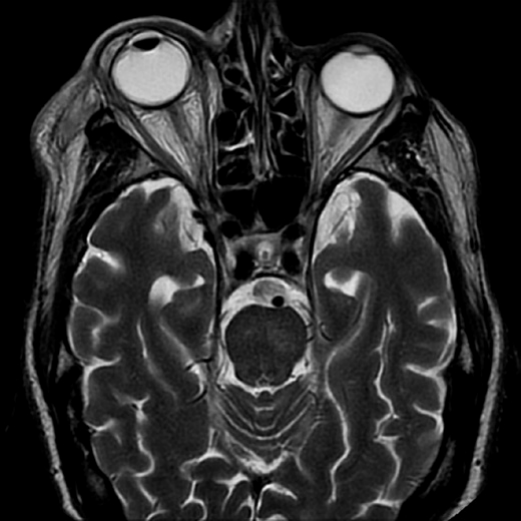
Magnetic resonance image showing proptosis of the right eye and increased difuse soft tissue volume, with T2 hyper intensity in both the pre- and post-septal spaces. These findings are compatible with orbital cellulitis and small subconjunctival abscesses.

In the ophthalmologic exploration, an amaurotic eye with areflexic myosis, conjunctival hyperemia, and intraocular pressure (IOP) of 46 mmHg was observed, without other significative changes. The patient was treated with intravenous (IV) 20% mannitol at 250 ml once and acetazolamide 250 mg orally every 8 hours with subsequent IOP lowering. A few hours later, a remarkable proptosis is observed, accompanied by chemosis, vascular ingurgitation, and “frozen eye”. Under the suspicion of a retro-bulbar hematoma or carotid-cavernous fistula, emergency magnetic resonance imaging (MRI) and angio-MRI were performed. No signs of vascular disorders were discovered, except for an increase in soft tissue both in the pre- and post-septal spaces, without paranasal sinus occupation, compatible with orbital cellulitis (Figure 1). In the blood count, neutrophilia and leukocytosis were present.

The patient had no previous history of trauma either on the affected or fellow eye. Prior to the described events, the patient had no vision complaints. The fellow eye was normal to exploration on portable slit-lamp examination, presented a normal IOP of 16 mmHg, and showed no abnormalities on funduscopic exploration. The presenting visual acuity (VA) of the affected eye was amaurosis (no light perception) from the moment the patient was first examined by the ophthalmologist. In the first visit, the VA of the fellow eye was not examined because the patient was in a bed in the stroke unit. When the patient was later transferred to our ophthalmology department, the fellow eye had a 16/20 VA.

On the basis of these results, conjunctival secretion samples were tested for bacteria and fungi. Blood cultures were taken, and new blood tests, including anti-nuclei antibodies, anti-neutrophil cytoplasmic antibodies, and angiotensin-converting enzyme, were requested. The infectious disease department was consulted, and broad-spectrum IV antibiotic treatment was initiated with piperaciline-tazobactam 4 g every 6 h and linezolid 600 mg every 12 h. Topical antibiotic treatment was also initiated with tobramycin 1 drop every 3 hours and ofloxacin 1 drop every 3 hours. IV steroids (methylprednisolone 60 mg every 24 hours) were also administered 48 hours after the start of the systemic antibiotherapy.

**Figure 2:**
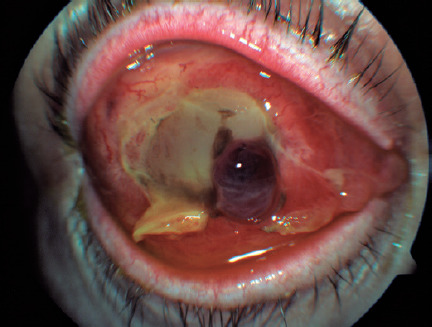
Corneal melting with tissue necrosis and intraocular content protrusion.

**Figure 3:**
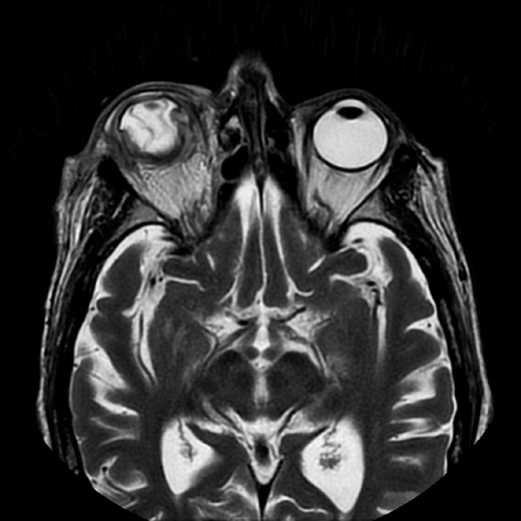
Orbital magnetic resonance image (MRI) showing decreased contrast of the pre- and post-septal soft tissues as compared that in the previous image, with destruction of the eyeball integrity, collapse of the anterior chamber, and retinal and choroid detachment.

Seventy-two hours from the onset of the symptoms, the RE developed an ischemic necrosis of the anterior segment that led to a spontaneous corneal perforation (Figure 2). A new orbital MRI was performed to search for an orbital or subperiostic abscess, without any findings suggestive of its presence (Figure 3).

The RE was eviscerated, and samples were sent to the pathology and microbiology departments. Necrotic tissue compatible with endophthalmitis and *Clostridium septicum* was isolated in the ocular remains and vitreous humor. The blood cultures and results of other blood tests (performed under the suspicion of an autoimmune origin, sarcoidosis, or Wegener granulomatosis) were negative. An extension study was requested to examine the endogenous origin of the infection. A mass in the transverse colon was detected on abdominal CT scan. Colonoscopy and posterior pathological examinations confirmed the presence of a colon adenocarcinoma.

## DISCUSSION

*Clostridium septicum* panophthalmitis is a rare form of gangrenous panophthalmitis with a typically torpid evolution. Even with precocious systemic broad-spectrum antibiotic treatment, to which the pathogen is sensitive, the affected tissue is still seriously damaged. The disease could cause sepsis and even lead to the death of the patient.

*Clostridium septicum* is a gram-positive bacterium that releases powerful exotoxins that destroy the surrounding tissues^(2)^. Death as a complication of panophthalmitis caused by *Clostridium septicum* infection has been described in the literature^(3,4)^.

A strong association was found between clostridium infection and colorectal carcinoma^(5,6)^. *C. septicum* proliferates in low oxidation-reduction potential conditions^(7)^. These circumstances do not occur in healthy intestines. They, however, occur in colorectal carcinomas, so a complete systemic study must be performed in these cases to find the origin of the infection^(8,9)^. In some cases, as in our case, the first manifestation of colorectal carcinoma is panophthalmitis^(10)^.

Owing to the aggressive nature of this infection, a precocious instauration of broad-spectrum antibiotherapy is key to a positive outcome. In our case, corneal perforation and loss of the eye could not be avoided, but evolution to sepsis or even death was eluded.
